# Changes in Actinomycetes community structure under the influence of *Bt* transgenic brinjal crop in a tropical agroecosystem

**DOI:** 10.1186/1471-2180-13-122

**Published:** 2013-05-29

**Authors:** Amit Kishore Singh, Major Singh, Suresh Kumar Dubey

**Affiliations:** 1 Department of Botany, Banaras Hindu University, Varanasi, 221005, India; 2 Indian Institute of Vegetable Research, Varanasi, 221305, India

**Keywords:** Actinomycetes, Community structure, Rhizosphere, *Bt* brinjal, *Cry1Ac* gene

## Abstract

**Background:**

The global area under brinjal cultivation is expected to be 1.85 million hectare with total fruit production about 32 million metric tons (MTs). Brinjal cultivars are susceptible to a variety of stresses that significantly limit productivity. The most important biotic stress is caused by the Brinjal fruit and shoot Borer (FSB) forcing farmers to deploy high doses of insecticides; a matter of serious health concern. Therefore, to control the adverse effect of insecticides on the environment including the soil, transgenic technology has emerged as the effective alternative. However, the reports, regarding the nature of interaction of transgenic crops with the native microbial community are inconsistent. The effect of a *Bt* transgenic brinjal expressing the bio-insecticidal protein (Cry1Ac) on the rhizospheric community of actinomycetes has been assessed and compared with its non-transgenic counterpart.

**Results:**

Significant variation in the organic carbon observed between the crops (non-*B*t and *Bt* brinjal) may be due to changes in root exudates quality and composition mediated by genetic attributes of *Bt* transgenic brinjal. Real time quantitative PCR indicated significant differences in the actinomycetes- specific *16S rRNA* gene copy numbers between the non-*Bt* (5.62-27.86) *×* 10^11^ g^-1^ dws and *Bt* brinjal planted soil (5.62-24.04) *×* 10^11^ g^-1^ dws. Phylogenetic analysis indicated 14 and 11, actinomycetes related groups in soil with non-*Bt* and *Bt* brinjal crop, respectively. *Micrococaceaea* and *Nocardiodaceae* were the dominant groups in pre-vegetation, branching, flowering, maturation and post-harvest stage. However, *Promicromonosporaceae, Streptosporangiaceae, Mycobacteriaceae, Geodermatophilaceae, Frankiaceae, Kineosporaceae, Actisymmetaceae* and *Streptomycetaceae* were exclusively detected in a few stages in non-*Bt* brinjal rhizosphere soil while *Nakamurellaceae, Corynebactericeae, Thermomonosporaceae* and *Pseudonocardiaceae* in *Bt* brinjal counterpart.

**Conclusion:**

Field trails envisage that cultivation of *Bt* transgenic brinjal had negative effect on organic carbon which might be attributed to genetic modifications in the plant. Changes in the organic carbon also affect the actinomycetes population size and diversity associated with rhizospheric soils of both the crops. Further long-term study is required by taking account the natural cultivar apart from the *Bt* brinjal and its near-isogenic non-*Bt* brinjal with particular reference to the effects induced by the *Bt* transgenic brinjal across different plant growth stages.

## Background

Brinjal (*Solanum melongena* L.) is the second largest vegetable crop in India reaching 8 to 9 million tons annually that amounts to one quarter of the global production, and is second to China [1]. It is a versatile crop that flourishes well under drought or salt stress. Insect pest infestations, however, limit the brinjal yield substantially [2]. It is susceptible to attack by many insect-pests, and more severely affected by the fruit and shoot borer (FSB). These insects effectively damage (60–70%) the crop even following the average 4.6 kg of insecticides and pesticides per hectare [2]. Therefore, to control the indiscriminate use of insecticides, the transgenic approach is being opted that is eco-friendly and shows promise to control the FSB infecting brinjal.

The use of insecticidal proteins from the bacterium *Bacillus thuringiensis* (*Bt*) in the improvement of crop productivity via transgenic crop (*Bt* crop) is being promoted in most cases. However, the potential risk associated with the impact of transgenic crops on non-target microorganisms and flora and fauna in the environment, is still a matter of concern. *Bt* crops have the potential to alter the microbial community dynamics in the soil agro-ecosystem owing to the release of toxic Cry proteins into the soil via root exudates [3], and through decomposition of the crop residues [4]. The available reports, however, are not consistent regarding the nature of interaction of transgenic crops with the native microbial community. Icoz and Stotzky [5] presented a comprehensive analysis of the fate and effect of *Bt* crops in soil ecosystem and emphasized for the risk assessment studies of transgenic crops. Phylogenetically, actinomycetes are the member of taxa under high G + C sub-division of the Gram positive bacteria [6]. Apart from bacteria and fungi, actinomycetes are an important microbial group known to be actively involved in degradation of complex organic materials in soils and contribute to the biogeochemical cycle [7]. The presence of *Micromonospora* in soils contributes to the production of secondary metabolite (antibiotics) like anthraquinones [8], and *Arthrobacter globiformis* degrades substituted phenyl urea in soil [9]. *Nakamurella* group are known for the production of catalase and storing polysaccharides [10]. *Thermomonospora,* common to decaying organic matter, are known for plant cell degradation [11]. *Frankia* is widely known for N_2_ fixation [12], *Sphaerisporangium album* in starch hydrolysis and nitrate reduction in soils [13], *Agromyces* sp. degrades organophosphate compounds via phosphonoacetate metabolism through catabolite repression by glucose [14]. *Janibacter* in rhizospheric soils, are widely known to degrade 1, 1-dichloro-2, 2- bis (4-chlorophenyl) ethylene (DDE) [15], while *Streptomyces* for the production of chitinase as well as antibiotics [16]. These studies suggest that most of the representative genera of actinomycetes in the soil, contribute to maintenance of the soil fertility.

Most studies on transgenic crops have been carried out on cotton, corn, tomato, papaya, rice, etc., with emphasis on protozoal, bacterial and fungal communities [5]. No information on the effect of transgenic brinjal on microbial community is available, though a few workers evaluated the influence of transgenic crops other than brinjal on actinomycetes based on population density using culture based CFU method (Additional file 1: Table S1) that has some limitations [17]. Rhizosphere is the most preferable ecological niche for microbial dynamics. It is a general assumption that rhizospheric microorganisms are the primary consumers of plant root exudates [18]. Therefore, it is expected that rhizospheric community dynamics will be affected by changes in the physiological activities of the plant as regulated by the genetic modifications induced. Considering above facts, the objective of this study was to assess the community structure (density and diversity) of actinomycetes associated with the rhizospheric soils of *Bt* transgenic brinjal. In addition, soil chemical properties were also determined as variations therein, are considered as the early indicators of the impact of transgenic crop on soil fertility [19].

## Methods

### Experimental site and crop description

Field trials were conducted in the agricultural farm of Indian Institute of Vegetable Research (I. I.V.R.), Varanasi, India (25° 08’ N latitude, 83° 03’ E longitude, 90 m from sea level, average temperature maximum 33°C and minimum 20°C). The site has been used for intensive vegetable production but not for any transgenic crop plantation prior to the present study (during 2010–2011). The soil (WHC 39.9%) is pale brown silty loam (sand 30%, silt 70%, clay 2%), Inceptisol with pH 6.7, organic C (0.73%) and, total N (0.09%) [20].

Ten- days old seedlings of VRBT-8 *Bt* transgenic event are selected for the study (data not shown). Genetic transformation was brought up through *Agrobacterium tumefaciens* LBA4404- mediated gene transfer that harbours pBinAR binary vector for neomycin phosphotransferase (*npt-II)* gene with neopaline synthase (NOS) promoter and a *Cry1Ac* gene fused to a constitutive, widely used plant promoter (CAMV35S) and octopine synthase gene (OCS) [21]. Treatments consisted of randomised blocks design in six plots of brinjal (*Solanum melongena* L. var. Kashi Taru), each 12 m^2^ (3 for transgenic -VRBT-8 and its near-isogenic non-transgenic, respectively) grown in containment condition to conform to bio-safety regulations and simulated agricultural conditions. Recommended cultivation practices were adopted in which soils prior to transplantation, were added with 25–30 tonnes/ ha farm yard manure (FYM) along with NPK (100–120 kg N, 75–85 kg P and 45–50 kg K) [22]. Irrigation was done at the interval of every 10–15 days to maintain optimum moisture conditions.

### Soil sampling and analyses

Soil sampling (in triplicate for each sampling stage) was done at different crop growth stages (branching, flowering and maturation) including pre-vegetation and post-harvest stage during the consecutive years (2010 and 2011). Rhizospheric soil samples were collected from the branching, flowering and maturation stage of non-*Bt* and *Bt* brinjal crop by uprooting the plants. Roots were vigorously shaken to separate the loosely bound bulk soils [23]. Soil samples at pre-vegetation and post-harvest stage, were collected from 0–10 cm depth using a 5 cm diameter soil corer [20]. To ensure the spatial homogeneity, soil samples were pooled and homogenously mixed prior to subsequent analyses. After removal of plant debris, samples were sieved through a 2-mm sieve and divided into two sub-samples. One sample was stored for 7 days (4°C) to prevent from sunlight and to reduce the microbial activity for molecular biological analyses (microbial density and diversity), and the other air dried for soil analyses.

Soil pH was determined using pH meter (Systronics-model 361). Organic carbon content was determined by wet digestion method of Walkey and Black [24]. The available Zn, Fe, and Mn in the soil samples were extracted with a diethylene triamine penta-acetic acid (DTPA) solution (0.005 M DTPA + 0.01 M CaCl_2_ + 0.1 M triethanolamine, pH 7.3 [25]. The respective micro-nutrients studied were Zn^2+^, Fe^2+^ and Mn^2+^. The available sulphur was determined using the method of Comb et al. [26], and available K_2_O by the method of Licina and Markovic [27].

### Soil DNA extraction

Total genomic DNA (in triplicate at each sampling stage) was extracted from 0.5 g rhizosphere soil using Fast DNA® spin kit (MP Biol, USA) combined with Fast DNA prep bead beater according to manufacturer’s protocol. The genomic DNA was eluted in 50 μl DNA eluting solution (DES) and stored (-20°C) for subsequent analysis. The concentration and purity of extracted DNA was determined using Nanodrop spectrophotometer (ND 1000, Nano Drop Technologies, Inc., Wilmington, DE, USA).

### Real time PCR for total actinomycetes *16S rRNA* gene copy number

Real Time Quantitative PCR (qPCR) amplification was performed using Applied Biosystems 7500 Fast Real –Time PCR system containing 96-well plate (ABI 7500) to quantify the abundance of total actinomycetes specific *16S rRNA* gene copy number using universal primer sets, 517 F (5’-CCA GCA GCC GCG GTA AT-3’) and Act704R (5’-TCT GCG CAT TTC ACC GCT AC-3’) [28]. The amplifications were carried out in triplicate in a final 25 μl volume containing 10X SYBR Green PCR master mix (Fermentas, USA). The reaction mixture (25 μl) comprised of 7.5 μl master mix (2X), 10 pmol each of primer (517 F and Act704R) and 45 ng genomic DNA template. The two-step Amp + Melt protocol was as follows: (i) amplification step: denaturing at 95°C for 4 min, 40 cycles of 30 s at 94°C and 30 s at 55°C, 1 min at 95°C, 1 min at 55°C, and (ii) melting curve analysis step: 81 cycles of 30s at 55°C. Plasmid DNA containing target gene (actinomycetes- specific *16S rRNA*) was used as the standard DNA in real time PCR assay, was obtained by PCR-cloning using the universal actinomycetes-specfic primers [28]. Standard curves were generated by plotting the threshold cycle for each standard, calculated with ABI Prism 7900 SDS 2.2.2 software (Applied Biosystem, USA), against the gene copy number. The amplification efficiency (E) was measured from the slope of the standard curve [29]. The standard curve revealed a slope of – 2.66 corresponding to an efficiency of 137. 39% and R^2^ of 0.994, similar to those reported in other studies [30].

### PCR amplification for actinomycetes-specific *16S rRNA* gene

Genomic DNA purified from soil was used as template for PCR. PCR triplicate from each sampling stages were separately amplified using universal actinomycetes-specific primers sets, ACT283F (5’-GGG TAG CCG GCC UGA GAG GG-3’) and 1360R (5’-CTG ATC TGC GAT TAC TAG CGA CTC C-3’) [12]. The PCR amplification was carried out using thermal cycler (Bio-Rad, USA) under the following conditions: (94°C, 5 min; 10 cycles of denaturation at 94°C (1 min), annealing at 65°C (30 s), extension at 72°C (2 min) and 72°C (5 min) followed by 20 cycles of denaturation at 92°C (30 s), annealing at 65°C (30 s), extension at 72°C (2.5 min) and final extension at 72°C (5 min). Reaction mixture (25 μl) contained 2.5 μl of 10 X buffer (Bangalore Genei, India), 0.5 μl of 40 mM dNTPs (Fermentas, USA), 1.25 μl each of 10 μM forward and reverse primer (Sigma), 2.5 U *Taq* DNA polymerase (Bangalore Genei, India.) and 1 μl template (40 ng). The remaining volume (18.5 μl) was maintained by nuclease-free water. Three PCR replicates of each samples stage were separately amplified and visualized on a 1.5% agarose gel. The resulting PCR products (1100 bp) were purified [31] through spin column using a QIAprep spin MiniPrep Kit according to manufacturer’s protocol, and combined separately for non-*Bt* and *Bt* samples.

### Cloning, restrction fragment length polymorphism and phylogenetic analyses

The purified PCR products were ligated into the p-GEM®T Easy vector at 4°C (Promega, USA) as per manufacturer’s protocol, and cloned into the CaCl_2_ treated *E.coli* DH5α competent cells. The screening of blue and white colonies was performed on ampicillin plates (100 μg ml^-1^) supplemented with X-gal (0.5 mM) and IPTG. A total of 350 clones (70 clones for each sampling stage) were checked for putative positive inserts by PCR targeted with plasmid specific primer M13 forward and M13 primers. Further details regarding the positive insert verification are as reported by Vishwakarma et al., [20]. The clones with insert showed amplification of more than 1300 bp, while the PCR products with lower bands (250 bp) corresponded to the plasmid vector without any insert.

To identify the unique, amplified insert, actinomycetes-specific clones were subjected to Restriction fragment Length Polymorphism (RFLP). Two actinomycetes-specific *16S rRNA*gene libraries were constructed, one for each soil actinomycetal community from the non-*Bt* plot and *Bt* brinjal plot. PCR products with inserts were used for producing RFLP pattern by digesting them with 0.4 U each of tetrameric endonuclease *Hha* I [30,32] and *Hae* III restriction enzymes (New England Biolabs, Beverly, MA) in 1X buffer B (New England, Biolabs), bovine serum albumin (10 mg mL^-1^) in the final volume of 20 μl. Reaction mixture was incubated overnight (37°C) and restriction patterns manually verified by analyzing 20 μl reaction mixture on 3% metaphor agarose gel stained with ethidium bromide in the gel documentation system (AlphaImager, NC, DE400-220, MTZ Zoom, Alpha Innotech Corp., San Leandro, CA). The zero–one matrices were prepared on the basis of RFLP pattern and operational taxonomic units (OTUs) grouped through CLUSTAL W program using the NTSYS version 2.1 software for each soil sample, and more than one representative of each group was sequenced.

The sequencing of the actinomycetal specific *16S rRNA* clones as performed on both the strands in ABI PRISM® 3100 Genetic Analyzer (ABI, USA) using the Big Dye Terminator Kit (Version 3.1). Electropherograms were generated using the Chromas freeware (Version 2.01; Chromas lite Technelysium Pvt Ltd, Australia). Clones were finally checked for chimeric artifacts using CHECK-CHIMERA of the Ribosomal Database Project, and the chimeric sequences were discarded. The *16S rRNA* sequences obtained, were initially recognized and aligned against the known sequences in the GenBank database using the BLAST program of the National Centre for Biotechnology Information (NCBI, http://www.ncbi.nlm.nih.gov/). The *16S rRNA* clones obtained from the non-*Bt* and *Bt* planted rhizospheric soils with > 90% similarity with the NCBI data base, were used for phylogenetic analysis using MEGA software version**.** Further details related to sequencing analysis are given elsewhere [33].

### Statistical analysis

The complete randomized design (CRD) with three replicates was used. Multivariate analysis of variance (MANOVA) was performed to determine the effect of treatments (non-*Bt* and *Bt*) at different growth stages. Multiple comparisons for difference in the means were made using Tukey’s Highest Significant Difference (HSD) test (P < 0.05), SPSS 16.0. The correlation coefficient was calculated between different parameters using the method given by Senedecor and Cochran [34]. The levels of significance (*P* < 0.01) and *P* < 0.05) are based on Pearson’s coefficients.

#### Nucleotide sequence accession numbers

The sequences of the *16S rRNA* gene reported in this study, have been deposited with the NCBI database under accession numbers: JQ285871- JQ285932.

## Results and discussion

It is well proven that plants affect the population and diversity of soil microbial communities, but the reports on the impact of transgenic crops on soil microbial communities, are contrasting. From (Additional file 1: Table S1 ), it is clear that transgenic crops affect the actinomycetes population. However, a few studies have focussed on the actinomycetes community structure [35-37]. Wei et al. [38] reported on the impact of transgenic papaya on soil macro- and micronutrients only during pre- and post-cultivations. The available information on the impact of transgenic crops during different crop growth stages is scanty. According to (Additional file 1: Table S2), the present study focussed on the impact of transgenic brinjal on the actinomycetes population size, community diversity and soil macro- and micronutrients throughout the vegetable crop cultivation cycle in two consecutive years ; a study not done before.

### Soil variables

Little but significant variation in the organic carbon as evident between the soil of non-*Bt* and *Bt* brinjal (Table 1), may be due to the changes in quality and quantity of root exudates mediated probably by genetic modifications in the brinjal crop [38]. The decreased activity of microbial biomass carbon (MBC) in the *Bt* brinjal planted soil could directly be linked with the reduction of organic carbon (data not shown). The availability and amount of organic carbon in soils is the key factor affecting activity and structure of the microbial community [39]. A slight change in the soil pH during the planting stages could probably be due to variations in the soil nutrient status and soil buffering capacity induced through the addition of chemical fertilizers along with the FYM [40]. From the present observations, it is clear that soil organic carbon is one of the important soil fertility attributes, and plays important role in the shifting of the microbial community in the rhizospheric of non-*Bt* and *Bt* brinjal crop.

**Table 1:** **Variation in soil pH, organic C (%), mineral-N (μg N g**^**-1**^**), K**_**2**_**O (kg hec**^**-1**^**), S (ppm), Zn (ppm), Fe (ppm) and Mn (ppm) in non-*Bt *and *Bt *planted soils Stages 1. Pre-vegetation; 2. Branching; 3. Flowering; 4. Maturation and 5. Post-harvest**

					**2010**				
**Stages**	**Crop**	**pH**	**Organic C**	**Mineral-N**	**K**_**2**_**O**	**S**	**Zn**	**Fe**	**Mn**
1	non-*Bt*	6.3 ± 0.11 ^a^	0.2 ± 0.12 ^a^	8.7 ± 0.57 ^a^	135.33 ± 7.85 ^a^	5.45 ± 0.03 ^a^	0.36 ± 0.03 ^a^	4.7 ± 0.20 ^a^	2.64 ± 0.29 ^a^
	*Bt*	6.3 ± 0.12 ^a^	0.2 ± 0.13 ^a^	8.7 ± 0.57 ^a^	135.33 ± 7.85 ^a^	5.45 ± 0.04 ^a^	0.36 ± 0.03 ^a^	4.7 ± 0.20 ^a^	2.64 ± 0.29 ^a^
2	non-*Bt*	6.86 ± 0.06 ^b^	0.59 ± 0.06 ^b^	14.64 ± 0.5 ^b^	169.6 ± 4.97 ^b^	6.10 ± 0.17 ^a b^	0.49 ± 0.03 ^b^	5.11 ± 0.01^a^	3.33 ± 0.39 ^b^
	*Bt*	7.03 ± 0.14 ^b^	0.47 ± 0.15 ^a^	15.53 ± 0.48 ^b^	156.5 ± 3.3 ^b^	5.8 ± 0.11 ^a b^	0.43 ± 0.01 ^b^	4.93 ± 0.24^a^	3.3 ± 0.13 ^b^
3	non-*Bt*	6.8 ± 0.06 ^b^	0.2 ± 0.16 ^a^	16.49 ± 0.39 ^c^	246.46 ± 2.02 ^c^	6.35 ± 0.08 ^b c^	0.56 ± 0.06 ^b^	5.15 ± 0.41 ^a^	3.5 ± 0.03 ^b^
	Bt	7.16 ± 0.31^b^	0.66 ± 0.17 ^b^	17.33 ± 0.41 ^c^	240.4 ± 2.02 ^c^	6.01 ± 0.05 ^b c^	0.53 ± 0.04 ^b^	5.06 ± 0.25 ^a^	3.47 ± 0.11 ^b^
4	non-*B*t	6.9 ± 0.06 ^b^	0.64 ± 0.18 ^a^	15.9 ± 0.69 ^c^	217.33 ± 3.38 ^d^	6.43 ± 0.26 ^b d^	0.51 ± 0.03 ^b^	6.12 ± 0.25 ^b^	3.94 ± 0.01 ^c^
	*B*t	7.14 ± 0.18 ^b^	0.55 ± 0.19 ^b^	16.94 ± 0.58 ^c^	223.23 ± 8.3 ^d^	6.21 ± 0.4 ^b d^	0.46 ± 0.02 ^b^	5.46 ± 0.08 ^b^	4.04 ± 0.10 ^c^
5	non-*Bt*	6.83 ± 0.08 ^b^	0.4 ± 0.20 ^a^	11.68 ± 0.54 ^d^	141.0 ± 9.31 ^a^	6.93 ± 0.7 ^c d^	0.47 ± 0.20 ^b^	4.93 ± 0.19 ^a^	3.20 ± 0.04 ^b^
	*Bt*	6.96 ± 0.13 ^b^	0.26 ± 0.21 ^b^	11.14 ± 0.46 ^d^	154.46 ± 10.6 ^a^	6.97 ± 0.18 ^c d^	0.41 ± 0.01 ^b^	4.73 ± 0.28 ^a^	3.24 ± 0.14 ^b^
					**2011**				
1	non-*Bt*	6.45 ± 0.05 ^a^	0.19 ± 0.02 ^a^	8.76 ± 0.69 ^a^	140.66 ± 3.8 ^a^	5.0 ± 0.15 ^a^	0.38 ± 0..01 ^a^	4.5 ± 0.03 ^a^	2.83 ± 0.49 ^a^
	*Bt*	6.45 ± 0.05 ^a^	0.19 ± 0.02 ^a^	8.76 ± 0.69 ^a^	140.66 ± 3.8 ^a^	5.0 ± 0.15 ^a^	0.38 ± 0..01 ^a^	4.5 ± 0.03 ^a^	2.83 ± 0.49 ^a^
2	non-*Bt*	6.73 ± 0.06 ^b^	0.58 ± 0.05 ^b d^	15.52 ± 0.36 ^b^	182.33 ± 8.19 ^b^	5.9 ± 0.15 ^b^	0.49 ± 0.02 ^b^	5.06 ± 0.12 ^a b^	3.25 ± 0.16 ^a b^
	*Bt*	6.93 ± 0.1 ^b^	0.54 ± 1.73 ^b d^	14.32 ± 0.73 ^b^	180.33 ± 11.31 ^b^	5.66 ± 3.27 ^b^	0.44 ± 0.02 ^b^	4.75 ± 0.48 ^a b^	3.4 ± 0.30 ^a b^
3	non-*B*t	6.86 ± 0.03 ^b^	0.69 ± 0.04 ^c^	17.04 ± 0.29 ^c^	246.0 ± 2.03 ^c^	6.03 ± 0.08 ^b c^	0.52 ± 0.05 ^c^	5.4 ± 0.15 ^b c^	3.3 ± 0.15 ^a b^
	*Bt*	7.16 ± 0.31 ^b^	0.61 ± 0.01^c^	16.98 ± 0.06 ^c^	245.56 ± 2.94 ^c^	6.0 ± 0.1 ^b c^	0.50 ± 0.02 ^c^	5.06 ± 0.53 ^b c^	3.5 ± 0.26 ^a b^
4	non-*Bt*	6.9 ± 0.05 ^b^	0.64 ± 0.02 ^c d^	15.29 ± 0.35 ^d^	220.0 ± 15.53 ^c^	6.5 ± 0.14 ^c^	0.50 ± 0.03 ^b c^	5.96 ± 0.12 ^c^	3.81 ± 0.03 ^b^
	*Bt*	7.0 ± 0.25 ^b^	0.56 ± 0.01 ^c d^	16.58 ± 0.45 ^d^	236.93 ± 4.00 ^c^	6.1 ± 0.32^c^	0.46 ± 0.04 ^b c^	5.56 ± 0.28 ^c^	4.1 ± 0.55 ^b^
5	non-Bt	6.96 ± 0.21 ^b^	0.51 ± 0.08 ^b d^	11.7 ± 0.27 ^e^	146.9 ± 11.5 ^a^	7.25 ± 0.16 ^d^	0.46 ±0.02 ^b^	4.7 ± 0.25 ^b a^	3.0 ± 0.11 ^a^
	*Bt*	6.83 ± 0.08 ^b^	0.27 ±1.73 ^b d^	11.64 ± 0.52 ^e^	152.3 ± 8.99 ^a^	7.08 ± 0.13 ^d^	0.4 ± 0.24 ^b^	4.63 ±0.23 ^b a^	3.36 ± 0.07 ^a^

Letters a, b, c, d and some where common indicate that soil attributes do not change significantly (P < 0.05 by Tukey’s HSD test), ± indicate standard errors of the means.

### Variation in actinomycetes population size between the non-*Bt* and *Bt* binjal crop

**Figure 1:**
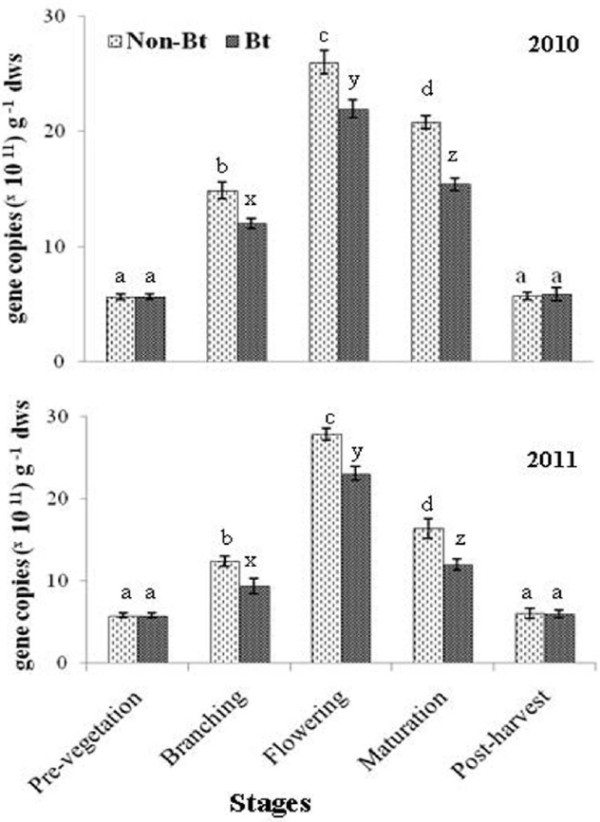
**Variation in actinomycetes population size in non-*Bt *and *Bt *rhizosphere soil at different crop growth stages in 2010 and 2011.** Different letter denote significant difference (P < 0.05) estimated by Tukey’s HSD, and the bar indicates extent of variation from the mean (n = 3).

Significant difference in the actinomycetes population between the soil of non-*Bt* and *Bt* brinjal over the entire two year period of cropping is depicted in Figure 1. Similar trend of variation in the actinomycetes population in the soil of non-*Bt* and *Bt* brinjal crop was: flowering > maturation > branching > post-harvest > pre-vegetation. Mean values for all the stages were significantly different from each other except between pre- and post-vegetation stages. MANOVA indicated significant differences due to year and crops (Table 2).

**Table 2:** Results of multivariate analysis of variance for observed parameters

	**2010**				**2011**				**Pooled**					
**Parameters**	**Stages**		**Crop**		**Stages**		**Crop**		**Year**		**Stages**		**Crop**	
	**F**_**(1,4)**_	**P**	**F**_**(1,1)**_	**P**	**F**_**(1,4)**_	**P**	**F**_**(1,1)**_	**P**	**F**_**(1,1)**_	**P**	**F**_**(1,4)**_	**P**	**F**_**(1,1)**_	**P**
Soil pH	6.50	0.002	2.20	0.153	8.73	0.000	0.52	0.599	0.45	0.504	14.59	0.000	3.34	0.075
Organic C	4.85	0.007	4.97	0.037	32.21	0.000	3.81	0.040	0.42	0.517	38.20	0.000	10.69	0.002
K_2_O	101.76	0.000	0.08	0.77	61.15	0.000	0.84	0.445	3.58	0.065	153.32	0.000	0.63	0.429
S	6.33	0.002	0.001	0.98	36.96	0.000	1.35	0.281	0.80	0.779	29.50	0.000	0.54	0.467
Zn	6.89	0.001	4.28	0.052	3.46	0.028	0.89	0.426	0.01	0.900	9.80	0.000	5.00	0.310
Fe	5.22	0.005	1.34	0.26	4.61	0.009	1.40	0.270	0.38	0.540	10.07	0.000	2.62	0.113
Mn	11.76	0.000	0.24	0.62	3.04	0.043	0.63	0.543	0.00	0.929	11.13	0.000	1.21	0.276
Mineral-N	88.16	0.000	1.73	0.202	96.06	0.000	0.81	0.458	0.03	0.847	182.7	0.000	0.92	0.347
Actinomycetes population	398.80	0.000	7.88	0.011	161.49	0.000	4.51	0.02	4.92	0.03	476.9	0.000	17.41	0.000

Tarafdar et al. [41] reported significantly higher actinomycetes population in non-*Bt* planted soil (5.25 X 10^6^ CFU g^-1^) compared to *Bt* brinjal planted soil (4.3 × 10^6^ CFUg^-1^). No significant changes were found in the studies conducted with transgenic cotton [42], corn [3], cabbage [43], and tomato [36]. Differences in the total actinomycetes population between the non-*Bt* and *Bt* crops might attributed to the release of root exudates from the transgenic brinjal into the soil that could have changed the available organic carbon and in turn, influenced the carbon turnover [38]. Tarafdar et al. [41] suggested that reductions in the actinomycetes population under *Bt* cotton cultivation were due to changes in the root exudates. However, other studies [3,36,44] supported that genetic modification of the plant had no role in changing the microbial population.

Significant differences in the actinomycetes population were observed between the crop growth stages (Table 2). Variation among the stages could be due to the changes in the soil nutrients e.g., available organic carbon, mineral-N, K_2_O, Zn, Fe, Mn and soil pH. The correlation analysis shows positive significant correlation of organic carbon content and mineral-N with population load of actinomycetes (r = 0.82, and r = 0.85 (Table 3), respectively). These results are consistent with those of others [45,46].

**Table 3:** Pearson’s correlation (r) matrix for soil pH, nutrients and actinomycetes population

**Properties**	**Year**	**Crop**	**Stages**	**pH**	**Organic C**	**K**_**2**_**O**	**S**	**Zn**	**Fe**	**Mn**	**Mineral- N**	**Actinomycetes population**
Year	1											
Crop	0.00	1										
Stages	0.00	0.00	1									
pH	-0.01	0.25	0.64**	1								
Organic C	0.58	0.24	0.52**	0.71**	1							
K_2_O	-0.21	0.21	0.02	0.62**	0.32	1						
S	-0.09	0.13	0.09	0.11	0.30	0.09	1					
Zn	-0.02	0.34	0.37	0.66**	0.93**	0.45**	0.40	1				
Fe	-0.98	0.24	0.35	0.52*	0.73**	0.11	0.25	0.67	1			
Mn	-0.00	0.14	0.54*	0.79**	0.71**	0.15	0.37	0.63**	0.81**	1		
Mineral-N	-0.00	-0.03	0.30	0.81**	0.92**	0.27	0.24	0.85**	0.74**	0.81**	1	
Actinomycetes population	-0.06	0.11	0.82	0.54**	0.82**	0.45**	0.04	0.84**	0.64**	0.56**	0.85**	1

** Correlation is significant at the 0.01 level (n = 20); * Correlation is significant at the 0.05 level (n = 20).

### Phylogenetic analysis of *16S rRNA* gene sequences from non-*Bt* and *Bt* brinjal rhizospheric soils

**Figure 2:**
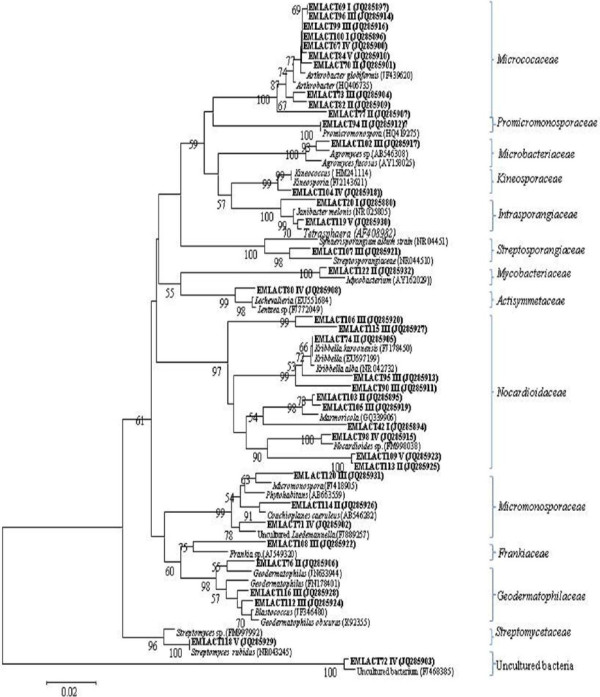
**Phylogenetic analysis of actinomycetes-specific *16S rRNA *sequences and related species by neighbor-joining method obtained from the non-*Bt *rhizosphere soil across the different crop growth stages.** Stages-I (Pre-vegetation), II (Branching), III (Flowering), IV (Maturation) and V (Post-harvest). Boot strap values above the 50% are indicated at the nodes. The scale bars represents 0.02 substitutions per site.

**Figure 3:**
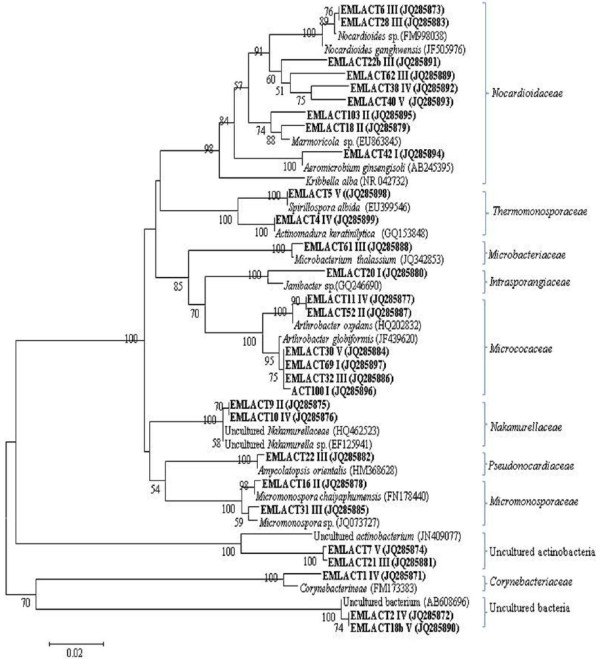
**Phylogenetic analysis of actinomycetes-specific *16S rRNA *sequences and related species by neighbor-joining method obtained from the *Bt *rhizosphere soil across the different crop growth stages.** Stages-I (Pre-vegetation), II (Branching), III (Flowering), IV (Maturation) and V (Post-harvest). Boot strap values above the 50% are indicated at the nodes. The scale bars represents 0.02 substitutions per site.

Thirty eight OTUs were generated from 282 positive clones for non-*Bt* brinjal soils. In case of *Bt* soils, a total of 278 positive clones clustered into 29 OTUs for pre-vegetation, branching, flowering, maturation and post-harvest stages. Different OTUs when evaluated after RFLP finger-printing analysis, showed affiliation with 14 and 11 actinomycetal groups from the respective non-*Bt* and *Bt* brinjal soils (Figure 2 and Figure 3). The percentage similarity of the clones with their reference strain as reported in the NCBI database is also shown in Table S3 and S4 (Additional file 1: Table S3 and S4).

**Figure 4:**
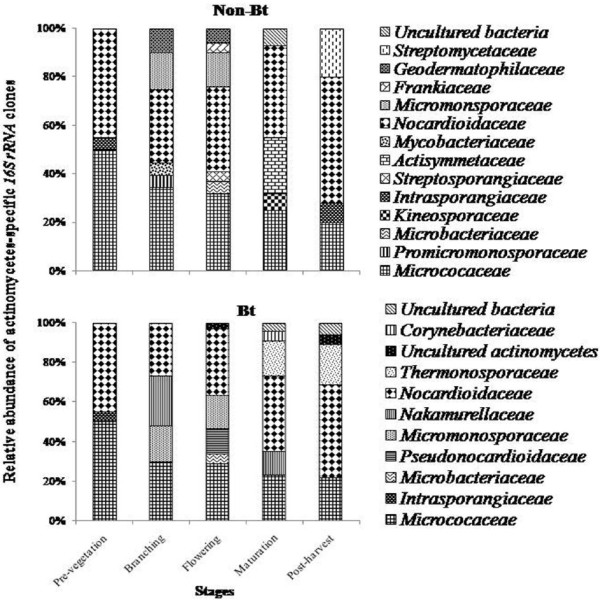
**Relative proportion of actinomycetes-specific *16S rRNA *clones across the different crop growth stages in non-*Bt *and *Bt *rhizosphere soil.**

Phylogenetic analysis indicated *Micrococaceae* and *Nocardioidaceae* as the dominant groups in the non-*Bt* and *Bt* cultivated soils as well. These respective groups were strongly represented by high relative abundance with > 40% of actinomycetes-specific 16S rRNA clones during each sampling stage (Figure 4). OTUs of these groups were affiliated with *Arthrobacter globiformis* (99%) and either of the *Nocardioides ganghwansis* (99%) or *Marmicola* sp. (98%), respectively (Table S3 and S4). In addition, *Intrasporangiaceae, Micromonosporaceae* and *Microbacteriaceae* were also detected in the non-*Bt* and *Bt* rhizospheric soils, but were restricted to only some of the sampling stages. *Intrasporangiaceae* was present at pre-vegetation stage with relative abundance of 5% (non-*Bt* and *Bt* soils), and in post-harvest stage (non-*Bt* soils only) raising the abundance to 8%. *Micromonosporaceae* was characterized by relative abundance (> 14%) at branching and flowering stage of non-*Bt* brinjal crop and with more than 17% abundance at the respective stages of *Bt* crop. However, *Microbacteriaceae* group was detected only at the flowering stage (relative abundance, 5%) in the rhizospheric soils of non-*Bt* and *Bt* crop (Figure 4). OTUs belonging to these respective groups showed their resemblance with *Janibacter* sp., *Micromonospora* sp., and either of *Agromyces* sp. or *Microbium thalassium* for *Microbiaceaea* (Table S3 and S4) that are mostly reported from soils. [8,15]

Groups like *Promicromonosporaceae, Streptosporangiaceae, Mycobacteriaceae, Geodermatophilaceae, Frankiaceae, Kineosporaceae, Actisymmetaceae* and *Streptomycetaceae* were exclusively detected for non-*Bt* while *Nakamurellaceae, Corynebactericeae, Thermomonosporaceae* and *Pseudonocardiaceae* for *Bt* rhizospheric soils, and were restricted to only some of the crop stages (Figure 2 and Figure 3). Both *Promicromonosporaceae* and *Mycobacteriaceaea* related OTUs were detected only during branching stages with relative abundance of 5%. OTUs of *Streptosporangiaceae* and *Frankiaceae* were present only during flowering stage (4% relative abundance) while that of *Geodermatophilaceae* were also present during branching in addition to the flowering stage with relative abundance of 10 and 6%. *Kineosporaceae* and *Actisymmetaceae* resembling OTUs were present only during maturation stage with 7 and 23% relative abundance, respectively. *Streptomycetaceae* group was confined to the post-harvest stage (20% relative abundance). *Nakamurellaceae* was detected during branching and maturation stages with 25 and 12% relative abundance, respectively while *Pseudonocardiaceae* only during flowering stage (12.5% relative abundance) (Figure 4). However, *Thermomonosporaceae* in addition to post-harvest stage (20%), was also detected during maturation stage (18% abundance) along with *Corynebacteriaceae* (5%). Except *Nakamurellaceae*, most of the OTUs of such exclusive groups of the non-*Bt* and *Bt* crop were affiliated with the reference strains that mostly originated from the soil / rhizospheric soil of the plants (Table S3 and S4).

In the present study, *Micrococaceae* and *Nocardioidaceae* were found to be the dominant group in cultivated soils. These taxa have been selectively enriched by the increased organic input to the soil [47,48], and also frequently detected in the manure and organic compost treated soils [49,50]. OTUs belonging to the exclusive groups in non-*Bt* and *Bt* planted soils as discussed above, are probably due to the specific nature of root exudates whose quantity and quality are likely to change via *Cry1Ac* gene based modification [3]. Rengel et al. [51] suggested that the resulting variations in the root exudates could be caused by the transformation of the plants. However, these exclusive actinomycetes groups were restricted to only a few growth stages of non-*Bt* and *Bt* crop. Also, the relative abundance of these OTUs for both the crops did not exceed the dominant taxa (*Arthrobacter* and *Nocardia*) as found for both the crops. Our findings corroborate with the result of Weinert et al. [52] wherein the genetic modification effect is more prominent only at the maturation stage compared to others in transgenic potato. Thus, it could be inferred that the genetic modification of brinjal using *Cry1Ac* gene, will have little impact on distribution of the dominant microbial groups (*Micrococaceae* and *Nocardiodaceae)*.

Under the control of constructive promoter, the transgene *Cry1Ac* was expressed in all parts of the transgenic brinjal plant, throughout the entire cropping period [21]. However, the transgene was detected only during the flowering stage in the rhizospheric soils of *Bt* brinjal (data not shown). Sims and Holden [53] reported 50% decrease in the insecticidal activity of the Cry1Ab protein during 1.6 days, and 90% decrease within 15 days. Various studies suggested rapid degradation of Cry proteins but the reports are mostly contradictory [5]. It can be argued that transgenic brinjal influences the rhizospheric community in case trans-gene product comes in contact with the rhizospheric microbial community. Furthermore, significant changes in the organic carbon can also be one of the important soil factors to cause temporal shifts in the actinomycetal community, since changes in the microbial community are correlated with organic carbon content [45]. Changes in the other soil variables (mineral-N, K_2_O, S, Zn, Fe, Mn and soil pH) with respect to plant-age [54], can also have significant role in the maintenance of the rhizospheric microbial community. The present study also supports the view that the extent of genetic modification depends on the plant type, transgenes, and the conditions prevailing [23].

Irrespective of the crop type, flowering stage harbours more diverse actinomycetes compared to others. Some studies suggested that the structure and function of rhizospheric microflora was affected by physiological activities of plant [18,55,56]. Therefore, flowering stage may be the favourable one for microbial proliferation due to the active release of root exudates [52,57]. Observations in the present study are in agreement with the fact that the natural factors other than genetic modification have strong bearing on temporal shifting of the microbial community including the actinomycetes [36]. We now can summarize that changes in the actinomycetal community structure are closely associated with environmental factors such as soil variables that may favour the optimal proliferation of actinomycetal community [30]. The *Cry1Ac* gene induced effect has the potential in shifting of the actinomycetal community although it is transient compared to the plant-age effect in the transgenic brinjal agroecosystem.

## Conclusions

Changes in the organic carbon content between the non-*Bt* and *Bt* planted soil can be attributed to alterations in the quality and composition of root exudates that could be regulated by the genetic modifications in the crop. Alteration in the organic carbon between the soils of non-*Bt* and *Bt* brinjal could be one of the possible reasons for the minor fluctuations in the actinomycetes population density and diversity, although the dominant groups (*Micrococaceaea* and *Nocardiodaceae*) were more prominent than the exclusive groups as detected in non-*Bt* and *Bt* brinjal planted soil during the crop duration. Since, the present study is confined to small scale field experiments that are not sensitive to detect anything other than large and obvious effects, the assessment of risks to biological diversity has to be conducted on a long-term and large-scale basis. Therefore, to assess the behaviour of transgenic line, there is need to include natural cultivar deployed by the local farmer, in addition to *Bt* and its near-isogenic *Bt* crop. Therefore, on the basis of evidences offered, it is concluded that the effect induced by the genetic transformation of brinjal if any, are minor and transient compared to those induced by the plant age that seems more prominent. The knowledge accrued from the present study, will certainly help in understanding the natural variability of actinomycetes community associated with the rhizosphere of transgenic and non-transgenic brinjal crops, and provide the base line information for further assessment of potential ecological risks of transgenic brinjal, and its commercialization.

## Competing interests

The authors declare that they have no any conflict of interest.

## Authors’ contributions

AKS was involved in all experimental work including manuscript writing. MS and SKD were designed the experiments and gave all inputs necessary for manuscript completion. All authors read and approved the final manuscript.

## Supplementary Material

Additional file 1: Table S1 Summary of the field trial studies on the impact of transgenic crops on soil actinomycetes community. **Table S2.** Reported results on the effect of transgenic crops on actinomycetes population and structure and micro- and macro nutrients in soil with respect to non-transgenic crops. **Table S3.** Nucleotide sequence BLAST results of actinomycetes-specific *16S rRNA* clones from non-*Bt*-brinjal soil. **Table S4.** Nucleotide sequence BLAST results of actinomycetes-specific *16S rRNA* clones of *Bt*-brinjal soil. Click here for file
