# Supplementary material for: Changes in Actinomycetes community structure under the influence of Bt transgenic brinjal crop in a tropical agroecosystem
Source: BMC Microbiol. 2013 May 29;13:122. doi: 10.1186/1471-2180-13-122 (PMC3671975; doi:10.1186/1471-2180-13-122)
Supplement: Additional file 1: Table S1 — Summary of the field trial studies on the impact of transgenic crops on soil actinomycetes community. Table S2. Reported results on the effect of transgenic crops on actinomycetes population and structure and micro- and macro nutrients in soil with respect to non-transgenic crops. Table S3. Nucleotide sequence BLAST results of actinomycetes-specific 16S rRNA clones from non-Bt-brinjal soil. Table S4. Nucleotide sequence BLAST results of actinomycetes-specific 16S rRNA clones of Bt-brinjal soil. [file 1471-2180-13-122-S1.doc]

**Supplementary data**

**Table S1. Summary of the field trial studies on the impact of transgenic crops on soil actinomycetes communities**

| **Experimental site / crop** | **Experimental conditions** | **Findings** | **References** |
| --- | --- | --- | --- |
| Agricultural farm of East  Marion, New York; *Bt* corn  (*Cry1Ab* gene) and non-*Bt*  Corn  Experimental field of  Oberviehhausen, Germany;  Transgenic potato (*gbss* gene)  and non-transgenic potato  Experimental field of Sun  Yat-sen University campus,  Guangzhou, China; Transgenic  papaya (mutant *Prsv* gene)  and non-transgenic papaya  Agricultural field of New  Zealand Institute, Canterbury  New Zealand; Transgenic potato  (*Maiganin II g*ene) and parent plant  Agricultural field of Hotala,  Maharashtra, India ; *Bt* cotton  (*Cry* gene) and non- *Bt* cotton  Agricultural field of Taiwan,  Taiwan; Transgenic resistant  tomato (*Cmvp* gene)  and wild type tomato as  control one  Indian Agricultural Institute, New  Delhi, India; Two cropping systems  Sole *Bt* cotton (*Cry* gene), cotton  + peanut) | Enumeration of  actinomycetes population  using serial dilution method,  Immunological assay;  Larvicidal assay  16S rDNA PCR- DGGE,  group-specific PCR of  16S rDNA of actinomycetes  fragments  Soil physico-  chemical properties;  Estimation of actinomycetes  population using Martin’s rose  bengal streptomycin agar  medium  Enumeration of  microbial population;  molecular analysis of  actinomycetes using DGGE  Total actinomycetes  population and diversity was  studied using Actinomycetes  isolation agar media for  CFUs count  Enumeration of total  actinomycetes population  using glycerol-yeast extract  agar; Actinomycetes specific  16S rDNA- DGGE profile  Dehydrogenase  activity; Enumeration of total  actinomycetes population  using Kusters agar medium | 2.3 x 10^5^ CFU g^-1^ soil in both  *Bt* and non-*Bt* corn. No significant  differences in the CFUs of  actinomycetes between non-*Bt* and  *Bt* rhizospheric soil  The rhizosphere of the  transgenic and non-transgenic  potato showed high similarity  at all the stages  Significantly higher CFUs of  actinomycetes by 0.80 times in  transgenic papaya; Transgenic  papaya could change  the actinomycetes population  No or minor effect was detected on  plant associated actinomycetes  diversity  No significant changes in the  population was observed  between *Bt* and non- *Bt* field  cotton; No adverse effect of *Bt*  cotton on actinomycetes  Total actinomycetes population  were 10^4^ CFUg^-1^ dws. No  significant difference in the  population load between transgenic  and wild-type tomato; No change  in community structure  Higher actinomycetes population  was estimated in peanut + transgenic cotton field (109.2 x  10^3^) CFU g^-1^ compared to *Bt*  cotton alone (98.9 x 10^3^) CFU g^-1^;  Application of urea and farm yard  manure mask the effect of *Bt* toxin  on the actinomycetes population | Saxena and Stotzky, (2001)  Milling *et al*., (2004)  Wei *et al.*, (2006)  Callaghan *et al*., (2008)  Kapur *et al*., (2010)  Continued...  Lin and Pan, (2010)  Singh *et al*., (2012) |
|  |  |  |  |
| Experimental field of National  Academy of Agricultural  Science(NAAS), Korea;  transgenic cabbage (*bar* gene) and  non-transgenic cabbage, | 16S PCR amplification;  Diversity analysis using DGGE,  enumeration of actinomycetes  population using R2A agar | 1.1 x 10^6^ - 4 x 10^6^ CFUg^-1^ fws  ranges were estimated in transgenic  and non-transgenic cabbages at  different sampling periods;  No significant difference in the  diversity and actinomycetes  population density were detected in  transgenic and non-transgenic  cabbage soil | Sohn *et al*., (2012) |
| Agricultural land of Vidarbha,  Maharashtra, India; *Bt* cotton  (*Cry 1Ac* gene) and  non-*Bt* cotton | Microbial C,N,P; Total  population of actinomycetes  and other microflora using  serial dilution and enzyme  activities | 52.5 x 10^5^ CFU g^-1^ in non-*Bt*  and 43.6 x 10^5^ in *Bt* cotton  soils. Significant reduction in  the actinomycetes population  attributed to changes in  root exudates | Tarafdar *et al*., (2012) |

**Table S2.** Reported results on the effect of transgenic crops on actinomycetes population and structure and micro- and macro nutrients in soil with respect to non-transgenic crops

| **Transgenic crops** | **Actinomycetes population** | **Actinomycetes community structure** | **Soil micro- and macronutrients** | **References** |
| --- | --- | --- | --- | --- |
| Brinjal | Significant difference in the population due to decrease in organic carbon | Transient changes in the community structure due to changes in root exudates | Significant difference in the organic carbon content due to *Cry1Ac* gene | Present study |
| Cabbage | No differences in the population between transgenic and non-transgenic cabbage soils |  |  | Sohn e*t al*., (2012) |
| Corn | No significant difference |  |  | Saxena and Stotzky, (2001) |
| Cotton | Significant reduction of population in *Bt* cotton soil due to changes in root exudates via genetic modification |  | No change | Tarafdar *et al*., (2012) |
| Cotton | No differences |  |  | Kapur *et al*., (2010) |
| Cotton | Few differences in the population attributed to cropping practice rather than the genetic modification |  |  | Singh *et al*., (2012) |
| Papaya | Higher population in transgenic papaya soils compared to non-transgenic soils due to increased Km-resistant microorganisms |  | Genetic modification affects the soil nutrients status up to some extent only | Wei *et al*., (2006) |
| Potato |  | No change |  | Milling *et al*., (2004) |
| Potato |  | Little effect of on the targeted community structure |  | Callaghan *et al*., (2008) |
| Tomato | No change | No change |  | Lin and Pan, (2010) |

|  | **Table S3** Nucleotide sequence BLAST results of actinomycetes-specific *16S rRNA* clones from non-*Bt*-brinjal soil | | | | | |
| --- | --- | --- | --- | --- | --- | --- |
|  | OTUs | Close NCBI BLAST match | Phylogenetic affiliations | Similarity (%) | | Habitat |
|  |  |  |  |  | |  |
| EMLACT69 I | | *Arthrobacter globiformis* | *Micrococaceaea* | | 99 | Soil |
| EMLACT96 III | | *Arthrobacter globiformis* | *Micrococaceaea* | | 99 | Soil |
| EMLACT99 III | | *Arthrobacter globiformis* | *Micrococaceaea* | | 99 | Soil |
| EMLACT100 I | | *Arthrobacter globiformis* | *Micrococaceaea* | | 99 | Soil |
| EMLACT67 IV | | *Arthrobacter globiformis* | *Micrococaceaea* | | 99 | Soil |
| EMLACT84 V | | *Arthrobacter globiformis* | *Micrococaceaea* | | 99 | Soil |
| EMLACT70 II | | *Arthrobacter globiformis* | *Micrococaceaea* | | 99 | Soil |
| EMLACT73 III | | *Arthrobacter globiformis* | *Micrococaceaea* | | 99 | Soil |
| EMLACT82 II | | *Arthrobacter globiformis* | *Micrococaceaea* | | 99 | Soil |
| EMLACT77 II | | *Arthrobacter globiformis* | *Micrococaceaea* | | 99 | Soil |
| EMLACT94 II | | *Promicromonospora* sp. JSM 099011 | *Promicromonosporaceae* | | 99 | Soil |
|  |  |  |  |  |  |  |
| EMLACT102 III | | *Agromyces* sp. IY07 | *Microbacteriaceae* | | 98 | Cool and subtropical soil |
|  |  | *Agromyces* sp. AR33 | *Microbacteriaceae* | | 97 | Rhizosphere soil |
| EMLACT104 IV | | Uncultured *Kineococcus* sp. LIM42 | *Kineosporaceae* | | 97 | Carbonate rock |
|  |  | *Kineosporia* sp. 65293 | *Kineosporaceae* | | 96 | Medicinal plant |
| EMLACT20 I | | *Janibacter* sp. M2T2B13 | *Intrasporangiaceae* | | 94 | Fermented and non-fermented bovine products and soil |
| EMLACT119 V | | *Tetrasphaera* sp. YC6726 | *Intrasporangiaceae* | | 98 | Rice field soil |
| EMLACT107 III | | *Sphaerisporangium album* | *Streptosporangiaceae* | | 96 | Forest soil |
| EMLACT122 II | | *Mycobacterium bacterium* | *Mycobacteriaceae* | | 97 | Soil |
| EMLACT80 IV | | *Lechevalieria* sp*.* C61 | *Actisymmetaceae* | | 99 | Hyper-arid soil |
|  |  | *Lentzea kentuckyensis* | *Actisymmetaceae* | | 98 | Mercury and chrome polluted soil |
| EMLACT106 III | | *Marmicola* sp. G soil 818 | *Nocardioidaceae* | | 98 | Environmental samples |
| EMLACT115 III | | *Marmicola* sp. G soil 818 | *Nocardioidaceae* | | 98 | Environmental samples |
| EMLACT74II | | *Kribbella karoonensis* | *Nocardioidaceae* | | 99 | Rhizosphere soil of cactus |
| EMLACT95III | | *Kribbella* sp. GTVB23 | *Nocardioidaceae* | | 99 | Acidic soil |
| EMLACT90 III | | *Kribbella alba* | *Nocardioidaceae* | | 99 | Soil |
| EMLACT103 II | | *Marmicola* sp. G soil 818 | *Nocardioidaceae* | | 98 | Environmental samples |
| EMLACT105 III | | *Marmicola* sp. G soil 818 | *Nocardioidaceae* | | 98 | Environmental samples |

Continued..

| OTUs | Close NCBI BLAST match | Phylogenetic affiliations | Similarity (%) | Habitat |
| --- | --- | --- | --- | --- |
| EMLACT42 I | *Marmicola* sp. G soil 818 | *Nocardioidaceae* | 98 | Environmental samples |
| EMLACT98 IV | *Nocardioides* sp. RS-51 | *Nocardioidaceae* | 99 | Rhizosphere soil |
| EMLACT109 V | *Nocardioides* sp. RS-51 | *Nocardioidaceae* | 99 | Rhizosphere soil |
| EMLACT113 II | *Nocardioides* sp. RS-51 | *Nocardioidaceae* | 99 | Rhizosphere soil |
| EMLACT120 III | *Micromonospora* sp. FXJ1.178 | *Micromonosporaceae* | 96 | Acidic soil |
|  | *Phytohabitans* sp. K11-0047 | *Micromonosporaceae* | 96 | Plant roots |
|  |  |  |  |  |
| EMLACT114 II | *Couchioplanes caerulens* | *Micromonosporaceae* | 97 | Cool and subtropical soil |
| EMLACT71 IV | Uncultured *Luedemannella* sp. | *Micromonosporaceae* | 98 | Soil |
| EMLACT108 III | *Frankia* sp. BMG5.5 | *Frankiaceae* | 95 | Soil |
|  |  |  |  |  |
| EMLACT76 II | *Geodermatophilus* sp. OS1-28 | *Geodermatophilaceae* | 97 | Rhizosphere soil of cactus |
| EMLACT116 III | *Geodermatophilus* sp. OS1-28 | *Geodermatophilaceae* | 97 | Rhizosphere soil of cactus |
| EMLACT 112 III | *Blastococcus* sp. FXJ6.383 | *Geodermatophilaceae* | 96 | Seawater, Sediments, Sponge |
|  | *Geodermatophilus obscurus* | *Geodermatophilaceae* | 96 | Dry soil, rocks, mountain surface |
| EMLACT118 V | *Streptomyces* sp. RP-B17 | *Streptomycetaceae* | 96 | Rhizosphere soil |
|  | *Streptomyces rubidus* | *Streptomycetaceae* | 96 | Acidic soil |
| EMLACT72 V | Uncultured bacterium | Uncultured bacteria | 92 | Agricultural soil |

**Table S4** Nucleotide sequence BLAST results of actinomycetes-specific *16S rRNA* clones of *Bt*-brinjal soil

| OTUs | Close NCBI BLAST match | Phylogenetic affiliations | Similarity (%) | Habitat |
| --- | --- | --- | --- | --- |
|  |  |  |  |  |
| EMLACT6 III | *Nocardioides* sp. RS -51 | *Nocardioidaceae* | 98 | Rhizosphere soil |
| EMLACT28 III | *Nocardioides ganghwensis* | *Nocardioidaceae* | 98 | Concrete surface |
| EMLACT22b III | *Nocardioides ganghwensis* | *Nocardioidaceae* | 98 | Concrete surface |
| EMLACT62 III | *Nocardioides ganghwensis* | *Nocardioidaceae* | 98 | Concrete surface |
| EMLACT38 IV | *Nocardioides ganghwensis* | *Nocardioidaceae* | 98 | Concrete surface |
| EMLACT40 V | *Nocardioides ganghwensis* | *Nocardioidaceae* | 98 | Concrete surface |
| EMLACT103 III | *Marmicola* sp. D13 | *Nocardioidaceae* | 97 | Nickel mine |
| EMLACT18 II | *Marmicola* sp. D13 | *Nocardioidaceae* | 97 | Nickel mine |
| EMLACT42 I | *Aeromicrobium gingsengisoli* | *Nocardioidaceae* | 98 | Gingsengisoli field soil |
| EMLACT5 V | *Spirillosa albida* | *Thermonosporaceae* |  | Thai cave soil |
| EMLACT4 IV | *Actinomadura keratinilytica* | *Thermonosporaceae* | 98 | Forest soil |
| EMLACT61 III | *Microbium thalassium* | *Microbacteriaceae* | 99 | Pepper plant soil |
| EMLACT20 I | *Janibacter* sp*.*M2T2B13 | *Intrasporangiaceae* | 94 | Fermented and non-fermented bovine products and soil |
| EMLACT11 IV | *Arthrobacter oxydans* | *Micrococaceae* | 99 | Mountain |
| EMLACT52 II | *Arthrobacter oxydans* | *Micrococaceae* | 99 | Mountain |
| EMLACT30 V | *Arthobacter globiformis* | *Micrococaceae* |  | Soil crusts |
| EMLACT69 I | *Arthobacter globiformis* | *Micrococaceae* | 99 | Soil crusts |
| EMLACT32 III | *Arthobacter globiformis* | *Micrococaceae* | 99 | Soil crusts |
| EMLACT100 I | *Arthobacter globiformis* | *Micrococaceae* | 99 | Soil crusts |
| EMLACT 9 II | Un*cultured Nakamurellaceae* | *Nakamurellaceae* | 98 | Environmental sample |
| EMLACT 10 IV | Uncultured *Nakamurella* | *Nakamurellaceae* | 99 | Biofilm reactor |
| EMLACT 22 III | *Amycolatopsis orientalis* | *Psuedonocardiaceae* | 99 | Soil |
| EMLACT 16 II | *Micromonospora chaiyaphumensis* | *Micromonosporaceae* | 99 | Rhizosphere soil of cactus |
| EMLACT 31 III | *Micromonospora* sp. NEAU-N1 | *Micromonosporaceae* | 99 | Soyabean root |
| EMLACT 7 V | Uncultured actinobacterium | Uncultured actinobacterium | 94 | Rhizosphere soil of cucumber |
| EMLACT 21 III | Uncultured actinobacterium | Uncultured actinobacterium | 93 | Rhizosphere soil of cucumber |
| EMLACT 1 IV | *Corynebacterium* | Uncultured actinobacterium | 97 | Tufa core sample |
| EMLACT 2 IV | Uncultured bacterium | Uncultured bacterium | 99 | Rice paddy soil |
| EMLACT 18b V | Uncultured bacterium | Uncultured bacterium | 98 | Rice paddy soil |
